# One Health in Indigenous Communities: A Critical Review of the Evidence

**DOI:** 10.3390/ijerph182111303

**Published:** 2021-10-28

**Authors:** Tamara Riley, Neil E. Anderson, Raymond Lovett, Anna Meredith, Bonny Cumming, Joanne Thandrayen

**Affiliations:** 1 National Centre for Epidemiology and Population Health, The Australian National University, Canberra 2601, Australia; raymond.lovett@anu.edu.au (R.L.); joanne.thandrayen@anu.edu.au (J.T.); 2 Roslin Institute, Royal (Dick) School of Veterinary Studies, University of Edinburgh, Roslin EH25 9RG, UK; neil.anderson@ed.ac.uk (N.E.A.); anna.meredith@unimelb.edu.au (A.M.); 3 Faculty of Veterinary and Agricultural Sciences, Melbourne Veterinary School, University of Melbourne, Parkville 3010, Australia; 4 Animal Management in Rural and Remote Indigenous Communities (AMRRIC), Darwin 0801, Australia; bonny.cumming@amrric.org

**Keywords:** One Health, indigenous health, animal health, environmental health, holistic, transdisciplinary

## Abstract

Indigenous populations around the world face disproportionately high rates of disease related to the environment and animals. One Health is a concept that has been used effectively to understand and address these health risks. One Health refers to the relationships and interdependencies between animal, human, and environmental health and is an emerging research field that aligns with indigenous views of health. To understand the applicability of One Health in indigenous communities, a critical review was undertaken to investigate evidence of One Health research in indigenous communities internationally, assess the strength of evidence, and understand what gaps are present. This review included the appraisal of twenty-four studies based in five regions: Canada, Africa, Australia, South America, and Central America. The review found that there is a need for studies of high strength, with rigorous methods, local leadership, and active involvement of indigenous viewpoints, to be undertaken in indigenous communities internationally that focus on One Health. It highlights the need to further consider indigenous viewpoints in research to reduce limitations, increase effectiveness of findings, consider appropriateness of recommendations, and benefit communities.

## 1. Introduction

The One Health concept can be traced back to the ancient Greek philosopher Hippocrates who recognized that human health depends on the environment [1]. However, it is likely that the ideologies surrounding One Health goes back further than this to indigenous societies and cultures that are tens of thousands of years old. The concept of One Health aligns with indigenous views encompassing a holistic view of health that recognizes traditional knowledge that links the health and wellbeing of animals, people, and the environment [2]. This approach is suitable in indigenous community settings as it aligns with cultural and community contexts, and indigenous ways of knowing, doing, and being. The One Health concept also considers health as more than the physical self, which is in line with the World Health Organization’s definition of health as ‘a state of complete physical, social and mental well-being, and not merely the absence of disease’ [3] (p. 1).

This holistic way of thinking continued into the nineteenth century with Rudolf Virchow stating that ‘between animal and human medicine there is no dividing line—nor should there be’ [4] (p. 100). In the 1980s ‘One Medicine’, which focused on bringing together the commonalities between animal and human medicine, was conceptualized by Calvin Schwabe [5]. In the early 2000s, following multiple zoonotic outbreaks, this concept evolved to ‘One Health’ which refers to a collaborative and interdisciplinary approach, at local, national, and global levels, to improve health for people, animals, and the environment [6]. There are similar concepts emerging from health sectors that have comparable aims with slightly different focuses. The ‘One Welfare’ concept has an emphasis on welfare; however, there is much overlap between these concepts, as One Welfare concentrates on the relationships between animal welfare, human wellbeing, and the environment [7]. The concept of ‘Ecohealth’ recognizes the inseparable linkages between ecosystems and the health of all species [8]. Similarly, the concept of ‘Planetary Health’ has also emerged and, driven through the human health sector, emphasizes human health systems and the linkages to political, economic, and social settings, and the Earth’s natural environment [9].

This review focuses on One Health which can be used as an approach to designing and implementing programs, policies, and research in which multiple sectors work together to achieve better public health outcomes [10]. Its benefits include more timely and effective responses, accurate decision making, accountability to other sectors, shared responsibilities and resources, and advocacy for policies and programs [10]. One Health is supported internationally through the World Health Organization, the World Organization for Animal Health, and the Food and Agricultural Organization of the United Nations Tripartite Alliance that promotes the concept of One Health as the optimal method of preventing and controlling emerging and endemic zoonotic diseases. This interdisciplinary approach has been noted as an effective way of addressing health threats at the human–animal–environment interface and is the best way of preventing and responding to future zoonotic outbreaks and pandemics [11]. One Health can also take a transdisciplinary approach, which can be particularly relevant when working in indigenous community contexts as this requires a ‘whole-of-society’ approach and recognizes cultural knowledge [12].

Research has shown that animal and environmental factors contribute to the health and wellbeing outcomes of indigenous peoples, including the ongoing impact of environmental stress on the health of animals and people [13,14]. Many public health risks that may benefit from a One Health approach disproportionally affect low socio-economic communities, many of which have high indigenous populations. Such risks include exotic zoonotic disease threats, such as an increased risk of rabies incursion into remote Aboriginal communities in Northern Australia [15]. This also includes endemic diseases (including neglected zoonotic diseases) that are of particular concern for low socio-economic communities [16]. Whilst zoonotic diseases may have significant impacts on communities, they are also among the most under-diagnosed diseases in humans, with the full burden of disease not well understood [16]. Within Aboriginal communities in Australia, there is a particular need for high-quality, large-scale, and comparative studies of companion animals and people from the same household to further assess zoonotic disease risk and prevalence [17]. While there is limited evidence in Australia, findings and recommendations internationally indicate that a One Health approach has the potential to improve health within indigenous communities [18,19,20].

The One Health field is still emergent. Public health interventions remain predominantly designed and managed in siloed health sectors, with limited communication and collaboration [8]. To date, the animal health sector has largely supported and promoted the One Health concept, with much of the literature and programs emerging through the animal health field [21]. Animal programs that deliver health care and consider a One Health approach are becoming more common internationally, particularly in low-resource communities. However, many of these programs do not incorporate traditional knowledge and teachings, with indigenous involvement commonly ceasing at the engagement phase [22]. A transdisciplinary approach is likely to improve this. When designing and implementing these programs, it is important to consider that animals have integral roles in indigenous communities and can be of cultural significance through traditional values and beliefs. In some Australian Aboriginal communities, dogs can be seen as the spiritual protector of the family and the land, and integrated into the kinship system [23,24]. They are also commonly accepted as family members and provide a sense of purpose for owners, with many owners feeling strong bonds and connections with their dogs [25]. Companion animals can also be helpful for hunting, providing protection, companionship, and warmth, and can provide health benefits for their owners through psychological, social, and emotional benefits, and improving health outcomes [26].

Given the significance and meaning of animals in indigenous communities and the benefits of animal companionship, ensuring the health and welfare of animals should be seen as a priority. However, many indigenous communities face barriers in accessing ongoing and effective animal health care [27]. Barriers can include limited resources and funding, remoteness and accessibility, limited veterinary services and animal medicines, and climatic impacts which differ between countries [20]. For example, many Australian Aboriginal communities face tropical monsoon climates that restrict access because of flooding, whereas Canadian Indigenous communities can be cut off by ice for much of the year [20]. Low socio-economic position, language, literacy and numeracy barriers, and awareness of the benefits of animal health care also limit the use of animal medicines and veterinary services where these are available [24]. Studies have found that community animal health and management programs can be beneficial in these settings, with community-specific strategies that take a transdisciplinary approach the most effective [28]. However, the evidence of a One Health approach including culturally appropriate programs incorporating local beliefs regarding the roles and importance of animals within the indigenous health space is limited.

This critical review aimed to understand One Health research that has been undertaken in indigenous communities worldwide, assess the strength of the evidence base, and identify what gaps exist. This review will be used to inform the development of a One Health model for use in Aboriginal communities with particular interest in improving animal health care and community health outcomes.

## 2. Materials and Methods

A critical review of the evidence provides an opportunity to understand and assess current evidence and frameworks and consider the development of a new model [29].

### 2.1. Indigenous Approach

This review was undertaken by an Australian Aboriginal-led interdisciplinary team that considered indigenous research methodologies, recognizing the cultural knowledge surrounding the interconnectedness between animal, human, and environmental health. Rigney described the importance of privileging indigenous voices as a key component of indigenous research methodologies [30]. Indigenous research methodologies recognize the impact of colonization and the political and social structures which continue to impact indigenous peoples [31]. Following this methodology, this review concentrated mainly on countries that have settler-colonial societies and indigenous populations which have been influenced by colonization, racism, and social injustice [31]. It also adapted an existing framework to include a focus on indigenous viewpoints in the scoring of evidence to assess the manner of indigenous involvement and priorities in the evidence base.

### 2.2. Search Strategy

Due to the differences in the use of terms relating to holistic health approaches globally, and the differing focuses of these approaches, multiple terms were included in the review. The definitions of the terms used were:

- Ecohealth: ‘Ecohealth is committed to fostering the health of humans, animals, and ecosystems and to conducting research which recognizes the inextricable linkages between the health of all species and their environments’ [9] (p. 3);
- One Health: ‘One Health is the collaborative effort of multiple health science professions, together with their related disciplines, and institutions—working locally, nationally, and globally—to attain optimal health for people, domestic animals, wildlife, plants, and our environment’ [9] (p. 2–3);
- One Welfare: ‘One Welfare describes the inter-relationship between animal welfare, human wellbeing and the physical and social environment’ [7] (p. 1);
- Planetary Health: ‘The achievement of the highest attainable standard of health, well-being, and equity worldwide through judicious attention to the human systems—political, economic, and social—that shape the future of humanity and the Earth’s natural systems that define the safe environmental limits within which humanity can flourish’ [9] (p. 4).

### 2.3. Literature Sources and Search Terms

A literature search was conducted using PubMed, the Web of Science, The Australian National University (ANU) library super search, and the Australian Indigenous Health Infonet search engines. The search terms were developed through an iterative process with input from all co-authors. The search terms used are presented in Table 1.

### 2.4. Selection of Studies

All documents selected from the search were screened by two reviewers (TR and JT), with 12 studies screened by a third reviewer (NA), from animal health, human health, and One Health backgrounds. Reviewers discussed conflicts until a consensus was reached with respect to inclusion criteria. Studies were screened by the third reviewer when there was uncertainty as to whether a study met the inclusion criteria. The studies identified through the search were assessed against these inclusion criteria:

- Original research;
- Written in English;
- Published between 2010 and 2020;
- Full text available;
- One Health, Ecohealth, Planetary Health, or One Welfare focus;
- Animal, human, and/or environmental health component (at least two of these);
- An indigenous group as the main human population of interest.

Studies that met all the criteria were included in the review, with additional references included based on authors prior knowledge of the literature. All studies were stored in Endnote, and records of the search and inclusion assessment were kept in Excel.

### 2.5. Summarising and Assessing the Strength of Evidence (SOE)

We summarized the current literature on One Health research in indigenous communities globally. Each study was summarized by the One Health sector focus (animal, human, and environmental health, or multiple) and key findings and recommendations. For the purpose of this review, we defined the One Health sectors as: animals referred to domestic animals and their health outcomes, human referred to the indigenous communities or peoples and their health outcomes, and environment referred to ecosystems and biodiversity factors including the physical environment, plants, wildlife, and invertebrates that live within an ecosystem. The analysis included calculating percentages to describe the characteristics of the evidence base including the One Health sector, Indigenous viewpoint, strength of evidence (SOE), and region.

We assessed the SOE based on two reviewers’ assessments of the studies (TR and JT). Reviewers assessed them independently and discussed conflicts until a consensus was reached. The review was undertaken using a modified version of the SOE framework developed by the Evidence-based Practice Centre, established by the US Agency for Healthcare Research and Quality (AHRQ), and based on the grading of recommendations, assessment, development, and evaluations (GRADE) approach [32,33,34]. The SOE framework involves assessing each study across five domains including study limitations, directness, consistency, precision, and reporting bias, with the studies scored as high, moderate, low or insufficient [33]. Given the focus on indigenous populations, studies were also assessed for an indigenous viewpoint [35] (Table S1). 

Where a domain was not applicable to the study, this was noted as such and was not included in the overall score. There was no evidence found that any of the domains are more important than the other and should be weighted differently. Therefore, we considered each domain equal and allocated the same weighting. Each domain met was added and the studies were scored as:

- 0 domains met = insufficient SOE;
- 1–2 domains met = low SOE;
- 3–4 domains met = moderate SOE;
- 5–6 domains met = high SOE.

## 3. Results

**Figure 1:**
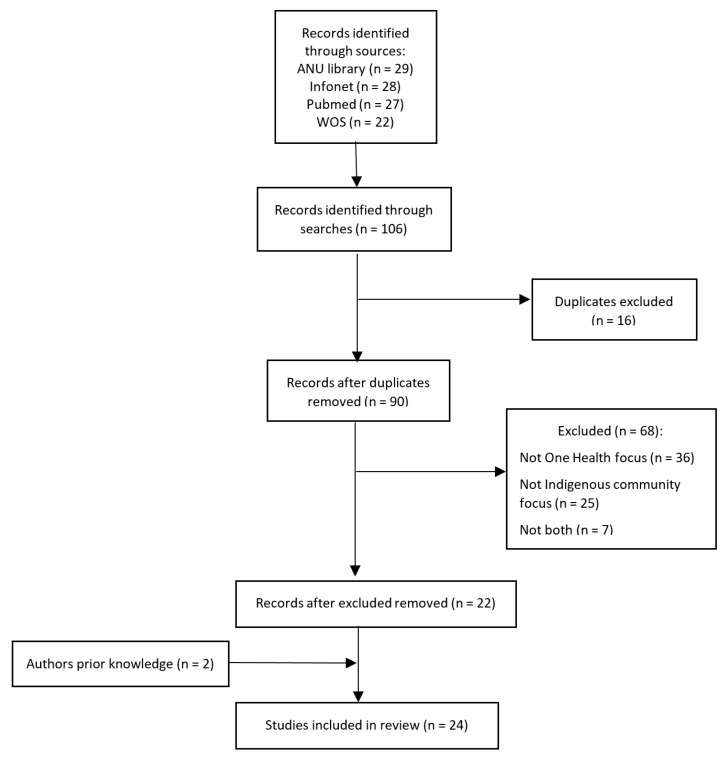
Inclusion flow chart.

The literature search identified a total of 106 peer-reviewed studies. After screening, 24 references (22 from the search and 2 from authors prior knowledge) were included in the analysis (Figure 1).

Overall, 46% of studies were rated as having a moderate SOE (*n* = 11), 29% were rated as low (*n* = 7), and 25% were rated as high (*n* = 6), with none rated as insufficient. The majority of studies incorporated all three One Health sectors (54%), followed by studies involving animals and people (33%), and studies involving people and the environment only (13%). The majority of studies had an indigenous viewpoint (19 of 24) including all studies scored as high, 91% of studies scored as moderate, and 43% of studies scored as low (Table 2).

Studies with a high SOE commonly had large sample sizes (for example, community level data or data from multiple communities), multiple sample types from different health sectors (for example, an animal and a human health variable), multiple time points for data collection (for example, repeated collection of data over multiple months or years), and indigenous viewpoints. The details of the evidence base and scoring can be seen in Table S2.

**Figure 2:**
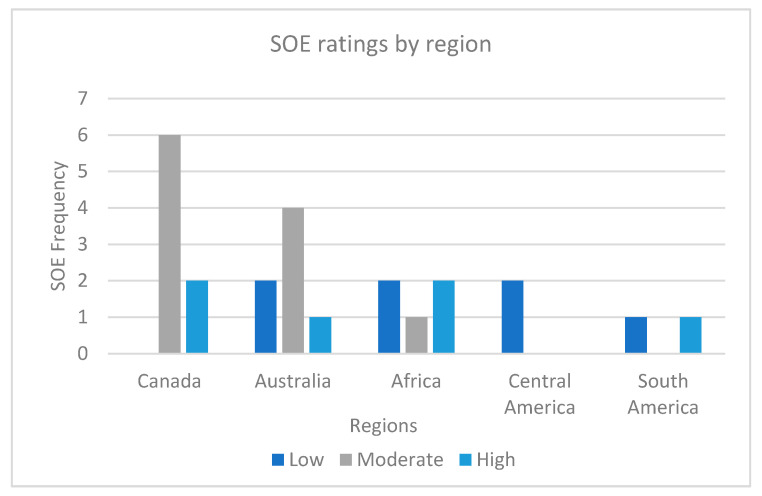
SOE ratings by region.

The main regions represented in the results were Canada (*n* = 8), Australia (*n* = 7), Africa (*n* = 5), Central America (*n* = 2), and South America (*n* = 2). Studies from Canada and Australia were most commonly scored as moderate, Africa and South America equally had studies scored as low and high, and Central America had studies scored as low. The frequency of SOE ratings in each region is shown in Figure 2.

## 4. Discussion

This review found that the incorporation of all three One Health sectors does not indicate a high SOE. Overall, studies were most commonly rated as having a moderate SOE (46%), followed by those scored as low (29%), and high (25%), with none scored as insufficient. While all studies were interdisciplinary, the majority included in the review incorporated all three One Health sectors (54%), followed by those involving animals and people (33%), and studies involving people and the environment (13%). As the search focused on indigenous communities, studies with a focus on animals and the environment only were unlikely to be identified which may have limited studies that incorporated an environmental health component. When broken down by SOE, 71% of studies scored as low included all three One Health sectors, compared to 45% of studies scored as moderate, and 50% of studies scored as high. Undertaking studies that incorporate all three sectors can be more complex compared to those which focus on two sectors only; however, they can also be more beneficial.

While the One Health concept commonly aims for interdisciplinary research, and this evidence base has demonstrated this, One Health within indigenous communities should strive for transdisciplinary research which takes a whole-of-society approach, values the inclusion of local community members, and prioritizes benefiting indigenous peoples [12]. While the concept has been supported by international health organizations, the practicalities of enacting it can be multifaceted and further consideration on how to truly implement the concept is needed [10]. The ownership and collaboration required between sectors can be a contested point, with many health sectors working within silos and limited examples of truly interdisciplinary work being operationalized and reported.

Environmental health is commonly under-represented within One Health [57] and this review supported this with environmental health the most common aspect missing. This is particularly pertinent for research undertaken with indigenous communities, as the environment plays an important role in connecting animal and human health, and can hold significant cultural meaning. Many of the studies assessed the connection between animal and human health and noted the lack of an environmental health component as a limitation. However, more than half of the studies did include all three One Health sectors which may reflect the high value placed on the environment by indigenous populations involved in the research. Social science is also commonly neglected in One Health research with the behavioral aspects of health and man-made environmental changes often not incorporated [26,58]. Therefore, ways to collaborate with the environmental health and social science sectors should be considered in future work [18,19].

The majority of the evidence base, and all high SOE studies, had an indigenous viewpoint; however, this was not always easy to assess with this domain having many limitations. We assessed a study as having an indigenous viewpoint if it involved community engagement, involvement, partnerships, or if it included indigenous authors; however, the latter was only possible when the authorship included information about the diversity of the authors. Studies were scored as having no indigenous viewpoint if it was not obvious whether an indigenous viewpoint had been sought. This approach was appropriate as indigenous research methodologies suggest the incorporation of community involvement and genuine engagement when undertaking research in the indigenous health space [31]. It is particularly relevant for One Health research to recognize and incorporate cultural beliefs and values which emanate around the connection to animals and the environment [59]. Following indigenous research methodology principles, the involvement of indigenous peoples within the research, including indigenous authors, should be sought and described explicitly to privilege indigenous voices [30].

Indigenous engagement is commonly stated as an aim of One Health research within communities; however, there is a lack of cultural knowledge and teachings incorporated into studies and programs delivered within communities [22]. Approaches to extend the involvement of indigenous peoples from engagement to partnerships and leaders within One Health is required to ensure this work is undertaken appropriately and for the benefit of communities. This is in line with indigenous research methodologies which recognize the importance and value of partnerships with community organizations and leadership by indigenous peoples [31]. It is important that this involvement is genuine and built on trust to ensure research is undertaken appropriately, incorporates community priorities, and recognizes cultural values and beliefs [59]. Additionally, indigenous-led research projects are being increasingly recognized as important worldwide as this allows indigenous peoples ownership over ideas, methodologies, and associated data, and can increase the effectiveness and sustainability of initiatives [60,61].

The regions included in the evidence base were Canada, Australia, Africa, Central America, and South America. Canada and Australia most commonly had moderately scored studies, with Africa and South America equally having studies scored as low and high, and Central American studies scored as low. This indicates that there is a need to increase the SOE of One Health research in all regions. Some regions were not represented in the analysis which may be due to holistic health and indigenous terms used in the search not being applicable in other regions, or studies not meeting inclusion criteria, such as written in English. It could also demonstrate a lack of research within these regions, demonstrating multiple gaps in this field internationally.

Strengths of this review included an interdisciplinary research team and the literature obtained from multiple sectors, taking a One Health approach to the review. This is also the first review that scores the evidence base of One Health in indigenous communities internationally. This review adopted an indigenous research methodology and modified an existing scoring framework to include an indigenously focused domain as part of the framework. This was important to the review as it focused on research related to indigenous communities around the world.

There were common study limitations through the evidence base related to sampling methods as many studies had convenience, opportunistic or referral sampling, used secondary data, had small sample sizes, or only involved participants from a biased sample, such as only animal owners. This restricted the ability to score the evidence and to some extent diminished the overall strength of the evidence base. Many of these studies were small and, therefore, it may not have been feasible to adopt a robust study design; however, the data and findings from these studies are still valuable to the evidence base. While there can be complexities of undertaking research with an interdisciplinary or transdisciplinary approach, there is a need to improve the evidence base by conducting research that involves robust, community level, primary data collection. However, the assessment criteria within this study were limited to a framework based on human health research and may need further adaption to meet the complexity of the One Health field. Another limitation noted in the evidence base was language barriers between researchers and participants, particularly in communities that spoke indigenous languages as the primary language. This highlights the importance of working with local people from the community to assist with translation and mitigate this limitation.

There was a small number of studies identified in the evidence base highlighting the gaps that exist in this field internationally; however, this is expected as One Health is an emerging research field particularly in relation to indigenous health. The search results were limited to studies written in English which may have introduced a geographical bias and excluded relevant studies written in another language. There was also differing usage of the holistic health and indigenous group terms between countries which may have further limited the results. The holistic health terms used are aiming to achieve similar outcomes and acknowledge the interconnection between animal, human, and environmental health; however, they all have slightly different focuses due to their emergence from different sectors [26]. There are reports of interventions within indigenous communities that aim to improve holistic health measures; however, these studies were not included in the review if they did not use one of the search terms. The assessment tool is also weighted heavily toward quantitative approaches to evidence. It is possible that it does not capture what qualitative elements are for evidence, and this could have affected the scoring of the evidence base. Many studies in the evidence base were prevalence, descriptive, or qualitative in nature, which were more subjective to assess and limited the ability to score the evidence. For example, if the study did not include an intervention, the directness of the study could not be scored, eliminating this domain and leading to a lower SOE. While this was common, no studies were excluded due to low SOE as they still contained valuable findings and recommendations.

For the One Health approach to be effective in indigenous communities, increased collaboration and communication between the animal and human health sectors is needed, with mutual recognition of the importance of the environment and ecological factors [5]. This may be particularly pertinent for indigenous health research due to the connections to the environment which many indigenous cultures have. The incorporation of social science to account for behavioral and social factors impacting health would also be helpful in producing comprehensive findings and recommendations [58]. Without this, we may fail to see the full benefits of a One Health approach. There is a need for One Health studies within indigenous communities internationally that have a high SOE and robust sampling methods. This finding is in line with prior studies that found a need for high-quality, large-scale, and comparative studies of companion animals and people from the same household in Australian Aboriginal communities [17]. Following indigenous research methodologies, the involvement and leadership of indigenous peoples in research within community settings should be prioritized to ensure consideration of community priorities and strengthen the evidence base [60].

While evidence shows that a One Health approach would be suitable in indigenous communities and is supported internationally, there is a lack of One Health models in indigenous communities described in the evidence base. Following recommendations from the review, a strong community One Health model for animal health care should focus on prevention and have indigenous leadership and involvement to be successful and sustainable. A transdisciplinary approach should be adopted to allow the incorporation of cultural knowledge and fit within indigenous community contexts and views of health that recognize the importance of animals and the environment to health. Increased collaboration and integration of community members into health systems and organizations would be beneficial to allow the sharing of knowledge and responsibilities between human, animal, and environmental health sectors and take a whole-of-community approach.

## 5. Conclusions

One Health is highly appropriate and applicable within indigenous communities as it adopts a holistic approach and is in line with indigenous cultural beliefs and views of health. However, this review found that there are limited One Health studies that have been undertaken in indigenous communities globally, with many gaps in the evidence base. There is a need for high SOE research to be undertaken in indigenous communities internationally to further understand how One Health approaches may be applied in this setting. The involvement and leadership of indigenous peoples in the research is vital to the effectiveness and sustainability of outcomes. The inclusion of environmental health needs further consideration to enhance understanding of the importance of the environment within health relationships. Increased collaboration and communication between health sectors are also needed to realize the benefits of One Health. This review recommends the development of an effective One Health model for animal health care within indigenous communities that is indigenous-led and transdisciplinary to address public health concerns and benefit communities.

## Figures and Tables

**Table 1:** Search terms.

Domain	Search Terms
Indigenous groups	Indigenous OR “First Nations” OR “First People” OR Aboriginal OR “Torres Strait Islander” OR “Native American” OR Inuit OR Maori OR Sami OR “Local tribe” OR “African tribe” OR Amazonian
One Health	“One Health” OR “One Welfare” OR Ecohealth OR “Planetary Health”
Animals	Animal *
Humans	Human * OR People * OR Person
Environment	Environment* OR Ecosystem*

* includes the plural form of the word in the search.

**Table 2:** SOE by One Health sector and indigenous viewpoint.

SOE	One Health Sectors N (%)			Indigenous Viewpoint N (%)		Total Studies N (%)	Corresponding Literature
	Human and Animal	Human and Environment	Human, Animal, and Environment	Yes	No		
High	2 (33)	1 (17)	3 (50)	6 (100)	0 (0)	6 (25)	[36,37,38,39,40,41]
Moderate	4 (36)	2 (18)	5 (45)	10 (91)	1 (9)	11 (46)	[13,15,20,42,43,44,45,46,47,48,49]
Low	2 (29)	0 (0)	5 (71)	3 (43)	4 (57)	7 (29)	[50,51,52,53,54,55,56]
Insufficient	0 (0)	0 (0)	0 (0)	0 (0)	0 (0)	0 (0)	
Total	8 (33)	3 (13)	13 (54)	19 (79)	5 (21)	24	

## Supplementary Materials

The following are available online at https://www.mdpi.com/article/10.3390/ijerph182111303/s1, Table S1: SOE framework domains and scoring; Table S2: Summary of literature and SOE.
