# Supplementary material for: One Health in Indigenous Communities: A Critical Review of the Evidence
Source: Int J Environ Res Public Health. 2021 Oct 28;18(21):11303. doi: 10.3390/ijerph182111303 (PMC8583238; doi:10.3390/ijerph182111303)
Supplement: Supplementary file 1 [file ijerph-18-11303-s001.zip › ijerph-1357172-supplementary.pdf]

One Health in Indigenous Communities: A Critical Review of the Evidence

Supplementary material

Table S1: SOE framework domains and scoring [32-35]

Domain	Scoring
Study limitations: degree to which a study is protected against bias. This domain was assessed through the study design (whether the design was appropriate) and conduct.	Low level of study limitations (coded as ✓)<br>Medium level of study limitations (coded as ×)<br>High level of study limitations (coded as ×)
Directness: whether the study links interventions directly to an outcome. Can include the direct comparison of interventions, populations, or outcomes within, and between, studies, and data provided directly from participants rather than through proxy respondents or secondary data.	Direct (coded as ✓)<br>Indirect (coded as ×)
Consistency: degree to which similar studies found the same direction of effect or outcomes, and how consistent the study is with others within the evidence base. This can be assessed by the direction or magnitude of effect.	Consistent (coded as ✓)<br>Inconsistent (coded as ×)<br>Unknown – single studies that do not have comparisons (coded as NA)
Precision: degree of certainty surrounding the direction of effect of a study outcome. This domain is based on the adequacy of the sample size and number of events. Examples of imprecise studies include those that use a small sample, a single time point, and those with wide confidence intervals or no statistical significance testing, affecting the quality of the data and findings.	Precise (coded as ✓)<br>Imprecise (coded as ×)
Reporting bias: publication, outcome reporting and analysis reporting bias. This domain assesses if studies selectively publish findings based on favourable outcomes or direction of effect, including not publishing adverse or non-significant findings, not reporting the full study details, or not reporting planned or unplanned outcomes depending on the results.	Suspected bias (coded as ×)<br>Undetected bias (coded as ✓)
Indigenous viewpoint: engagement and involvement of the community in the research, and community viewpoints on how the research was performed and perceived.	Yes (coded as ✓)<br>No (coded as ×)

Table S2: Summary of literature and SOE

Reference	*One Health sectors*			Focus	Summary	Recommendations	Study design	*SOE criteria*						SOE
	Human (Indigenous group)	Animal (domestic)	Environment (Inc. wildlife)					Study limitations	Directness	Consistency	Precision	Reporting bias	Indigenous viewpoint	
Aenishaenslin et al. 2019 (41)	One Inuit community, Nunavik, Canada	Dogs	NA	Understanding health and wellbeing impacts of animals	This study investigated factors that affect human and dog health, dog-related human health risks, and perceptions of dogs in Inuit communities.<br>Results showed different perceptions and practices related to dogs between Inuit and non-Indigenous people, and highlighted the positive role of dogs and their importance for Inuit health and wellbeing.	Use knowledge gained to develop integrated, sustainable and culturally adapted solutions for dog health and dog-related human health risks.<br>Repeat in multiple communities with more inclusion of non-dog owners.	Multi-disciplinary sequential explanatory mixed methods approach. Cross-sectional survey of 67 dog owners and interviews with 21 dog owners and non-owners.	×	NA	✓	×	✓	✓	Moderate
Aenishaenslin et al. 2014 (42)	Inuit communities, Nunavik, Canada	Dogs	Wildlife – arctic and red foxes	Rabies cases, exposures and vaccinations	This study investigated the distribution of rabies in Nunavik from 1999 to 2012.<br>Results found 44 cases of rabies mainly in arctic and red foxes, the number of dogs vaccinated was highly variable between regions, and 112 people had consultations for potential rabies exposure. Children <10 years were most commonly exposed and the median time between exposure and treatment was four days.	Follow a One Health approach to prevent rabies in humans in similar contexts worldwide.<br>Maintain and reinforce the dog vaccination program in this region, enhance surveillance on rabies particularly in wildlife, implement dog population control programs and improve education on dog bites and rabies.	Quantitative descriptive analysis of data by year and region from 1999 to 2012, using data from animal rabies tests and confirmed cases, dog vaccinations, and human consultations for rabies exposure.	×	NA	✓	✓	✓	×	Moderate
Anthonj et al. 2019 (35)	Kenyan Ewaso Narok Swamp, wetland communities, Africa	Livestock	Water related infectious disease (sanitation and poor hygiene, insect	Risk perception versus actual risk of infectious disease exposure	This study assessed risk perceptions towards infectious disease exposure and evaluated if perceived risks reflect actual risk.<br>Results found that risk perception reflects actual risk, with overall level of	Use local knowledge on infectious disease risk and take an integrated approach to health promotion and wetland management taking into account humans,	Mixed methods approach using a cross-sectional survey of 400 households (including smallholders, farmers,	✓	✓	NA	✓	✓	✓	High

Reference	*One Health sectors*			Focus	Summary	Recommendations	Study design	*SOE criteria*						SOE
	Human (Indigenous group)	Animal (domestic)	Environment (inc. wildlife)					Study limitations	Directness	Consistency	Precision	Reporting bias	Indigenous viewpoint	
			vectors, flooding and drought)	in wetlands	risk perception factors high. Risk factors included limited access to water, sanitation and poor hygiene, agricultural irrigation, proximity to livestock, use of agrochemicals, and weather events.	ecology and animals involved. Repeat as longitudinal study to assess risk in different seasons and years.	pastoralists, workers), and in-depth interviews with 20 community members from four communities.							
Baker et al. 2020 (20)	Four remote communities in Sahtu Settlement Area of North West Territories, Canada	Dogs	NA	Dog health program and community perception	This study evaluated a decade-long veterinary program in four remote communities to understand changes in dog husbandry and community perceptions since the start of the program in 2008. Results found significant increase in dog health indicators including preventative medicine and desexing. Owners commonly described dogs as part of the family, however communities differed in how they housed and fed dogs, and their attitudes towards them. This study shows that a one size fits all approach is not suitable in this context and community specific programs should be considered.	One size fits all approach is not suitable in this context and community specific programs should be considered. Regular evaluations are needed to ensure programs are meeting community needs and are effective. Repeat using focus groups or interviews instead of questionnaires. Further exploration of purpose and role of dogs, and changes in dogs' diets, as may have implication on health.	Questionnaire of 114 households and a dog census in four communities in 2008 and 2017. Statistical significance testing used to compare 2008 to 2017.	✗	✗	✓	✓	✓	✓	Moderate
Bardosh et al. 2014 (36)	Kilombero and Ulanga districts, Southern Tanzania, Africa	Dogs	NA	Community perception and responses to dog	Tanzania recently embarked on a rabies vaccination program where they vaccinated dogs in 28 districts. This study aimed to understand community perceptions	Interventions for neglected zoonotic diseases need to more explicitly engage with project organisation, capacity and	Over three months in 2012, 32 focus groups in 16 villages, 16 semi-structured	✓	✓	✓	✓	✓	✓	High

Reference	*One Health sectors*			Focus	Summary	Recommendations	Study design	*SOE criteria*						SOE
	Human (Indigenous group)	Animal (domestic)	Environment (inc. wildlife)					Study limitations	Directness	Consistency	Precision	Reporting bias	Indigenous viewpoint	
				rabies program	and responses to this program, and the relationships between dogs, society, geography, and project implementation. Results found that vaccination coverage in six villages was well below official estimates and that reluctance to participate was due to fear of rabies, high medical costs, and threat of dog culling, with differences between towns, rural and pastoralist populations.	community participation. Greater attention to navigating local realities in planning and implementation is needed to ensure zoonotic diseases are controlled sustainably.	interviews with village leaders, 19 semi-structured interviews with district officials and vets, questionnaires of 113 households, and population-based survey in six villages (n=6157).							
Beknazarova et al. 2020 (49)	27 remote Aboriginal communities, North and Central Australia	Dogs	Environmental faecal samples of unknown origin	Environmental and zoonotic sources of Strongyloides stercoralis and hookworm	This study investigated the prevalence of zoonotic S.stercoralis and hookworm in dogs and maps their distribution. Results found that 21.9% of dog samples had S.stercoralis and 31.4% had A.caninum with no other hookworm species found.	Use a One Health approach to inform future intervention and prevention strategies aimed at controlling these parasites in both dogs and humans.	274 dog faecal samples and 11 faecal samples of unknown origin (assumed to be from dogs) were collected from 27 sites from 2016 to 2019. They were tested for S.stercoralis and four hookworm species using PCR.	✗	NA	NA	✗	✓	✗	Low
Bevilacqua et al. 2015 (37)	Amerindian communities of Venezuela	NA	Insects, rainfall, temperature	Ecohealth framework developed for eliminati	This study used an adaptive management and Ecohealth framework for malaria elimination in communities in Venezuela. The framework was used to capture, organise and communicate connections	Consider opportunities to enact local capability, needs, and freedom of choice into this framework. Use an Ecohealth approach, with a focus on local	Mixed methods study. Records for rainfall, temperature and landscape analysed. Longitudinal studies	✓	✓	NA	✓	✓	✓	High

Reference	*One Health sectors*			Focus	Summary	Recommendations	Study design	*SOE criteria*						SOE
	Human (Indigenous group)	Animal (domestic)	Environment (inc. wildlife)					Study limitations	Directness	Consistency	Precision	Reporting bias	Indigenous viewpoint	
				on of malaria	and relationships among key factors related to malaria.	involvement and capability, for effectiveness and sustainability of the program.	conducted in three villages with species identification undertaken. Historical malaria data analysed from 1995-2012 and focus groups held.							
Blaizot et al. 2020 (50)	One Remote Amerindian community, Amazon	Cats	Water, soil	Investigation of an outbreak of acute toxoplasmosis	This study investigated an outbreak of acute toxoplasmosis in a remote village which is thought to be linked to wild cats and the rainforest ecosystem. Results found 20 cases of T.gondii in people, with one infected foetus dying, and all other cases mild. Domestic cats had recently appeared in the village and the quality of the drinking water was found to be unsafe. T.gondii was found in one water sample and nine soil samples, and the meat samples.	Public health action was recommended such as safe water supply, health advice, and surveillance of toxoplasmosis. A One Health strategy involving medicine, vets and public health should be pursued for better understanding of transmission routes and emergence of zoonosis.	Between May and July 2017, nine households (n=54) were tested for T.gondii using PCR. Samples of water, cat faeces, cat rectal swabs, soil and meat were tested. Positive PCR samples were genotyped.	×	NA	NA	✓	✓	×	Low
Brookes et al. 2020 (51)	Five remote Aboriginal communities in North Australia	Dogs	NA	Community perception of dog management policy and practice	This study explored how dog-related problems arise and are managed. Findings show free roaming dogs have broader impacts on canine and human health than previously documented. Enabling roaming can enhance wellbeing and family connectivity, however can cause	Environmental Health Workers (EHW) can provide supportive structure for dog owners and help manage the health and population of free roaming dogs. Use community based management practices to support dog health.	Participatory qualitative approach. Over a year, in-depth interviews with service providers and informal semi-structured discussions with community members were	×	NA	NA	×	✓	✓	Low

Reference	*One Health sectors*			Focus	Summary	Recommendations	Study design	*SOE criteria*						SOE
	Human (Indigenous group)	Animal (domestic)	Environment (inc. wildlife)					Study limitations	Directness	Consistency	Precision	Reporting bias	Indigenous viewpoint	
					disengagement and conflict.		undertaken (n=53).							
Cott et al. 2018 (43)	Four First Nations communities, Mackenzie River, North Canada	NA	Burbot fish (lota lota), organic pollutants and metals	Traditional methods of assessing fish as a food source	Burbot livers are a traditional food source in North Canada, however, there has been concern about the quality and potential contaminants. This study collected Burbot livers and ranked them using a traditional method of assessment.<br>This study found that traditional methods effectively assessed the quality of livers with those ranked most palatable also the most nutritious and safest. While copper was higher in those that were more palatable, it was still well below consumption guidelines.	Traditional methods of assessment could be incorporated into community-based monitoring frameworks as a rough index of overall fish and ecosystem health, and detection of environmental change.<br>Linking traditional knowledge into scientific studies and repeating this study overtime can help assess how environmental changes are affecting Burbot fish.	136 adult burbot were collected between Oct and Dec in 2007 and 2008, from traditional fishing areas. Burbots were assessed and ranked by experienced community fishermen. Livers were then tested in a laboratory and findings compared.	✗	✓	NA	✓	✓	✓	Moderate
Degeling et al. 2018 (15)	Aboriginal communities in East Arnhem Land (EAL) and Northern Peninsular Area (NPA) and Cairns, North Australia	Dogs	Wildlife – dingos	One Health approach to zoonotic disease control using rabies as an example	This study explored what AUSVETPLAN rabies response measures were justified or unacceptable and why, and what other steps need to be taken to prevent rabies. Findings show the biosecurity response is limited by structural disadvantage including poor infrastructure, appropriate information and engagement, dominant cultural norms, and food security concerns.	Efforts should be made to adapt interventions to local contexts and community preferences.<br>Communities at greatest risk need help to prepare and develop strategies to manage the biosecurity response to rabies.	Qualitative study with five collaborative workshops and 28 semi-structured interviews held between January 2017 and June 2018 in Cairns, NPA and EAL communities.	✗	NA	NA	✓	✓	✓	Moderate
Fiorello et al. 2017 (52)	Mayangnaand Miskitu	Hunting dogs	Wildlife – jaguars	Health of hunting dogs and	This study investigated the health of hunting dogs in three communities. Dogs	Better understanding of dog depredation by jaguars, pathogen	78 hunting dogs examined, and samples	✗	✓	NA	✗	✓	✗	Low

Reference	*One Health sectors*			Focus	Summary	Recommendations	Study design	*SOE criteria*						SOE
	Human (Indigenous group)	Animal (domestic)	Environment (inc. wildlife)					Study limitations	Directness	Consistency	Precision	Reporting bias	Indigenous viewpoint	
	people in three communities in Nicaragua Bosawas Biosphere Reserve			spillover to wildlife and humans	in all communities were nutritionally compromised and had a heavy burden of disease. Seroprevalence found more than 50% positive for canine distemper, parvovirus, rickettsia, and leptospira, and many dogs were anaemic and/or dehydrated. It was hypothesised that we may see disease spillover from hunting dogs to wildlife, particularly jaguars that are common in these areas and come into contact with dogs often.	exposure in jaguars, and management strategies for hunting dogs is needed to mitigate threats to jaguars, improve health of hunting dogs, and protect the health of their owners. Future studies should use PCR rather than serology to find active infections.	collected from 77. Surveys conducted with 19 dog owners using an interpreter.							
Gabriele-Rivet et al. 2019 (44)	Hunters from remote Aboriginal communities in Northern Peninsula Area (NPA), Queensland, North Australia	Dogs	Wild dogs	Hunters knowledge of zoonotic health risks from dogs	This study explored dog hunting practices, the domestic-wild dog interface, and intercommunity interactions of hunting dogs. The study found most hunters were not aware of diseases that could be transmitted to dogs or humans from wild animals. More than half had a wild dog encounter in the last year leading to identification of high risk areas in the NPA.	There is a need for educational programs on disease management in relation to hunting in Indigenous communities.	Cross sectional survey of 13 hunters on demographics, hunting practices and encounters with wild dogs delivered in Nov 2016 and March 2017. Social network mapping between hunters was undertaken.	×	NA	✓	×	✓	✓	Moderate
Hampson et al. 2011 (53)	Maasai pastoralists, Serengeti, North-West Tanzania	Livestock	Soil, climatic events, wildlife	Associations between animal and environmental exposure	This study explored anthrax records in the Serengeti in humans, domestic animals and wildlife. Anthrax was detected annually in numerous species, with large outbreaks spatially	Further explore the ecological and behavioural interactions to understand the variable patterns of infection and exposure among	Spatial and temporal case-detection and seroprevalence data from wild and domestic animals analysed.	×	NA	NA	×	✓	✓	Low

Reference	One Health sectors			Focus	Summary	Recommendations	Study design	SOE criteria						SOE
	Human (Indigenous group)	Animal (domestic)	Environment (Inc. wildlife)					Study limitations	Directness	Consistency	Precision	Reporting bias	Indigenous viewpoint	
				s of anthrax and disease	localised affecting herbivores. Soil alkalinity and weather extremes were predictors of spatial and temporal exposure and infection risk.	species. Use soil alkalinity and weather extremes to guide greater risk-based surveillance and management of anthrax, including preventative activities.	Investigated spatial, environmental, climatic and species-specific associations in exposure and disease.							
Harper et al. 2015 (13)	Rigolet Inuit community, Nunatsiavut, Labrador, Canada	NA	Climate change	Explore climate-health relationships and identify and rank climate sensitive health priorities	The study examined climate change impacts on local health and ranked climate sensitive health priorities. The climate sensitive health priorities for the region included food security, water security, mental health and wellbeing, new hazards and safety concerns, and health services.	The climate sensitive health priorities are applicable to the region and more research and planning should be undertaken using an Ecohealth community-based approach. Further research to differentiate the impacts of long term versus short term climate change impacts is needed.	Exploratory, participatory mixed methods study including in-depth interviews with regional health reps (n=11), two community surveys (n=187) and three photo voice workshops (n=11).	✗	NA	NA	✓	✓	✓	Moderate
Kansiime et al. 2015 (45)	Local communities in 3 sub-counties in Kiruhura District, Uganda	Livestock	NA	Brucellosis disease and prevention program perceptions	This study explored community perceptions on the integration of animal vaccination and health education by veterinary and public health workers in the management of brucellosis. All groups had a lack of awareness of brucellosis or vaccination. Community members noted that mass animal vaccination and education is important and government support is needed. Community participation was also	Stakeholders including wildlife authorities, community members, local and national political leaders, vets, human doctors and the public health sector should be involved. Community participation is vital in any further work. Future research could investigate the role of wildlife in the	Qualitative study with six focus groups (n=57) held with community members, local leaders and farmers, and five key informant interviews held with public health workers and vets.	✗	NA	✓	✓	✓	✓	Moderate

Reference	*One Health sectors*			Focus	Summary	Recommendations	Study design	*SOE criteria*						SOE
	Human (Indigenous group)	Animal (domestic)	Environment (inc. wildlife)					Study limitations	Directness	Consistency	Precision	Reporting bias	Indigenous viewpoint	
					noted as crucial for sustainability and ownership of the process.	epidemiology of brucellosis.								
Queenan et al. 2017 (54)	Maasai of Ngorongoro District, Tanzania	Livestock	NA	Local language surveillance for zoonotic disease	This study aimed to identify terms used to describe infectious diseases in livestock and humans that have the greatest impact on livelihoods, and characterise the usefulness and limitations of using local terminology. Zoonotic conditions and syndromes included brucellosis, emboroo, oloirobi, rabies, Tuberculosis and emburuo. Study found that several of the descriptive, syndromic local terminology terms were used inconsistently and showed temporal and spatial variations.	There is a need for further analysis, including lab diagnosis, to improve specificity before incorporating local terms into diseases surveillance systems and disease control programs.	Qualitative cross sectional study including semi-structured interviews with 23 groups (n=117) within five villages. Informal discussions and field observations were conducted with village elders, vets, medical officers, meat inspectors and livestock field officers.	×	NA	NA	×	✓	✓	Low
Riley et al. 2020 (38)	Remote Aboriginal community, Northern Territory, Australia	Dogs and cats	NA	Evaluation of animal health and management program	This study evaluated an animal health program over 12 months. It assessed animal health (desexing, body condition and hair score) and human health (dog bite) outcomes before versus after one year of program implementation. Animal health outcomes significantly improved and with no significant change in the human health outcome.	Incorporate community specific approaches and involve community members in programs for sustainability and effectiveness. Consider how to further incorporate human and environmental health aspects into the program for future One Health programs.	Quantitative evaluation of an animal health program using a pre-post design from June 2018 to June 2019. Annual household census of animals and quarterly health clinic data on dog bites analysed.	×	✓	✓	✓	✓	✓	High

Reference	*One Health sectors*			Focus	Summary	Recommendations	Study design	*SOE criteria*						SOE
	Human (Indigenous group)	Animal (domestic)	Environment (inc. wildlife)					Study limitations	Directness	Consistency	Precision	Reporting bias	Indigenous viewpoint	
Roegner et al. 2019 (55)	Mayangna and Miskitu people, Nicaragua remote Bosawa Biosphere reserve, North Caribbean	Hunting dogs	Wild game	Exposure to neglected tropical diseases by hunting dogs	This study explored if hunting dogs increased the risk of human exposure to protozoal zoonotic neglected tropical diseases (NTD).<br>Findings show Giardia was detected in 22% of faeces, including both zoonotic and dog specific genotypes, with shedding more common in older dogs and those in accessible communities. Cryptosporidium was not detected and Trypanosoma was detected in 9%. This study suggests that hunting dogs can serve as sentinels of environmental and zoonotic NTDs.	Future research should look to expand the number and diversity of villages sampled.	In 2013, dogs from three communities were examined. Dog faeces was screened for Giardia and Cryptosporidium DNA (n=58) and serum was screened for Trypanosoma (n=78).	×	NA	NA	×	✓	×	Low
Schurer et al. 2015 (46)	Three First Nations communities in Canada	Dogs	NA	Experiences of dogs and vet services in communities	This study aimed to document First Nations perspectives on dogs, and educate rural health workers on animal management services in communities.<br>Three digital stories were developed including desexing clinics, the roles of dogs, the human-animal bond, and healthy dogs as part of healthy communities. The stories saw changes in dog husbandry, acceptance of desexing programs, knowledge transfer between residents, researchers and policy makers, and an overall	Community engagement and consultation in veterinary services for dogs in First Nations communities is important. Cultural beliefs and positive stories of dog programs should be used to guide other programs.	Qualitative participatory study. 10-14 residents and healthcare workers took dog related photos and participated in group interviews during summer of 2014. Audio-visual data was synthesised into four digital stories.	×	NA	✓	✓	✓	✓	Moderate

Reference	*One Health sectors*			Focus	Summary	Recommendations	Study design	*SOE criteria*						SOE
	Human (Indigenous group)	Animal (domestic)	Environment (inc. wildlife)					Study limitations	Directness	Consistency	Precision	Reporting bias	Indigenous viewpoint	
					desire to continue dog programs.									
Schurer et al. 2013 (39)	Dene community, North Saskatchewan, Canada	Dogs	Wildlife	Assessing risk factors and parasite burden before and after intervention	This study looked at zoonotic parasite prevalence in animals and humans and the associated risk factors. After administration of pyrantel pamoate there was reduced shedding of T.canis and other roundworms. 65% of people tested were positive for at least one zoonotic parasite including 14% Toxoplasma gondii, 48% Echinococcus granulosus, 13% Toxocara canis and 16% for Trichinella. The survey indicated few associations between risk factors for parasite exposure and serological status. Only 17% of participants knew that pathogens could move between animals and people.	This study demonstrates the potential benefits of canine de-worming as a public health intervention and emphasised the importance of environmental transmission of parasites through soil, food and waterborne routes.	Faecal analysis of owned and free-roaming dogs was undertaken over two years (n= 92, n= 103). Administration of pyrantel to 122 dogs in the first year and ELISA tests of 201 people. A survey asking about associations between risk factors and exposure was also undertaken.	✗	✓	✓	✓	✓	✓	High
Schurer et al. 2015 (40)	Two First Nations communities, North Saskatchewan, Canada	Dogs	Wildlife	Evaluation of a community action plan designed to improve animal and human health	This study reported the effects of a community action plan designed to improve animal and human health, increase animal health literacy, and benefit community wellbeing in communities where a dog-related child fatality had occurred. Surveys showed low levels of veterinary intervention with 92% of dogs sexually intact, 6% vaccinated and	Ongoing surveillance of dog bites, parasite levels and dog demographics are needed to measure the benefits to dogs, humans and wildlife.	Mixed methods study. In 2013, stakeholder meetings and door-to-door demographic surveys were undertaken pre and post vet clinic. Elder tea groups (sharing circle), parasite analysis before and after	✓	✓	✓	✓	✓	✓	High

Reference	*One Health sectors*			Focus	Summary	Recommendations	Study design	*SOE criteria*						SOE
	Human (Indigenous group)	Animal (domestic)	Environment (inc. wildlife)					Study limitations	Directness	Consistency	Precision	Reporting bias	Indigenous viewpoint	
					6% wormed. There were approximately three animal related injuries requiring medical care per 1000 people per year reported and 86% of faeces analysed contained parasites. Following the program, parasite levels in dogs decreased significantly.		treatment, and dog bite surveillance data was analysed.							
Smout et al. 2017 (47)	Aboriginal communities, Far North Queensland, Australia	Dogs	Soil	Prevalence of hookworm in soil and free-ranging dogs	This study investigated the prevalence of A.ceylanicum in soil and free-ranging domestic dogs. Results found prevalence of hookworm was high (96.3% and 91.9%) with majority A.caninum species. The presence of A.ceylanicum was found in domestic dogs (21.7%) and soil (55.6%). A.ceylanicum was found in two out of three locations in the soil.	Ongoing dog health and management programs employing local Aboriginal residents are needed. Incorporating the diagnosis and treatment of disease in humans and dogs for more targeted and cost effective health programs. Dog health needs to be addressed as part of the Australian Government's commitment to addressing chronic disease in Aboriginal communities.	In 2011, six locations were sampled including three Aboriginal communities and three tourist attractions. 130 soil samples, 86 faecal samples from free-roaming dogs, and 27 samples from dog carcasses were analysed for hookworm using PCR.	✗	NA	NA	✓	✓	✓	Moderate
Tsou et al. 2018 (48)	Aboriginal communities, Midwest Western Australia, Australia	Animal management	Housing, pest control	Assessing the health community assessment tool (HCAT)	HCAT assesses environmental health conditions and social determinants of health in communities. This study assessed the applicability of the HCAT, its suitability, and its role in improving the environment. Participants assessed the tool and domains including	Youth engagement in education was of high concern and the development of a youth indicator would be useful. Further research in other communities to enable comparison of common issues is needed. Applying the	In 2013, five focus groups (total n=17 participants) were held in April and May (one in 2015) to inform and assess the tool. The tool was trialled in	✗	✓	NA	✓	✓	✓	Moderate

Reference	*One Health sectors*			Focus	Summary	Recommendations	Study design	*SOE criteria*						SOE
	Human (Indigenous group)	Animal (domestic)	Environment (Inc. wildlife)					Study limitations	Directness	Consistency	Precision	Reporting bias	Indigenous viewpoint	
					pest control and animal management, healthy housing, food supply, community vibrancy pride and safety, reducing environmental tobacco smoke, and promoting physical activity. The tool was found mostly suitable, however, some youth indicators were missing.	tool nationally would allow identification of state variations and similarities, and differentiate between policy outcomes.	communities and then adapted based on feedback and ratings.							
